# Assemblages and Subassemblages of *Giardia duodenalis* in Rural Western, Kenya: Association with Sources, Signs, and Symptoms

**DOI:** 10.1155/2024/1180217

**Published:** 2024-02-05

**Authors:** Erick Barasa, Briston Indieka, Nathan Shaviya, Ezra Osoro, Geofrey Maloba, Denis Mukhongo, Valentine Budambula, Tom Were

**Affiliations:** ^1^Department of Medical Laboratory Sciences, Masinde Muliro University of Science and Technology, P. O. Box 190-50100, Kakamega, Kenya; ^2^Department of Biomedical Science and Technology, Maseno University, Postal address, Private Bag Maseno, Kenya; ^3^Department of Medical Biochemistry, Masinde Muliro University of Science and Technology, P. O. Box 190-50100, Kakamega, Kenya; ^4^Department of Biological Sciences, Alupe University, P. O. Box 845-50400, Busia, Kenya; ^5^Department of Environment and Health, Technical University of Mombasa, Mombasa, Kenya; ^6^Department of Laboratory Medicine and Human Pathology, Masinde Muliro University of Science and Technology, Kakamega, Kenya

## Abstract

**Background:**

*Giardia duodenalis* causes sporadic or epidemic infections in humans. The parasite comprises assemblages A-H with A and B subdivided further into AI-IV and BI-IV subassemblages. Attempts aimed at linking these genotypes with sources and gastrointestinal manifestations of the infection are largely unexplored in rural communities.

**Methods:**

In this cross-sectional study, *G. duodenalis* infection was genotyped and associated with sources, and gastrointestinal signs and symptoms of the disease among residents of Busia County, a rural setting in western Kenya. Demographic and clinical information were captured using standardized forms. Stool specimens were obtained from the patients and used for genotyping at *glutamate dehydrogenase* and *triose-phosphate isomerase* loci using the polymerase chain reaction and restriction fragment length polymorphism.

**Results:**

Assemblage B (63.6%) was the most prevalent *G. duodenalis* infection, while A (20.5%) and mixed A/B (15.9%) were also detected. Among the subassemblages, AI (5.7%), AII (8.0%), AIII (3.4), BIII (30.7%), and BIV (17.0%) were diagnosed including the mixed AII/BIII (15.9%), BIII/BIV (15.9%), AI/AIII (2.3%), and AI/AII (1.1%) infections. Binary logistic regression indicated associations for assemblage A with stomach upset, history of nitroimidazole treatment, and residing in a homestead with cattle and B with age < 18 years, history of eating outdoors, vomiting, steatorrhea, and residing in a homestead with cattle, goats, and poultry (*p* < 0.05 for all). Among the subassemblages, associations were found for AI with residing in a homestead having cattle and history of nitroimidazole treatment, BIII with residing in a homestead having cattle and poultry, and BIV with steatorrhea (*p* < 0.05 for all). Altogether, this study illustrates that *G. duodenalis* assemblage B and subassemblage BIII are the most predominant and are linked to age < 18 years, gastrointestinal manifestations, and living in a homestead with domestic ruminants and poultry.

**Conclusion:**

Targeted mass prophylactic treatment of domestic animals and utilization of gastrointestinal presentations, age < 18 years, and a history of nitroimidazole use are useful in the diagnosis and prevention of giardiasis among residents of rural communities.

## 1. Introduction

Giardiasis is an intestinal protozoal disease caused by *G. duodenalis* and transmitted mainly through the faecal-oral route. Both humans and animals are affected with the World Health Organization reporting up to 200 million human infections in the world, largely in low-to-middle-income countries [1]. In sub-Saharan Africa, the infection rates of giardiasis in humans range from 1.3 to 39.9%, with higher rates of infection among children < 18 years and residents of rural regions [2]. The prevalence of giardiasis in Kenya ranges from 5.9 to 13.1%, with a majority of the cases being children and individuals in rural and informal settlements [2]. However, integrated water, nutrition, and sanitation intervention including floor type trials among residents of Kakamega, Bungoma, and Vihiga counties in western Kenya reported infection rates of 39.0% and 38.7% [3, 4], respectively, suggesting a heavy burden of giardiasis in rural western Kenya.

*Giardia duodenalis* consists of assemblages A-H with A and B frequently causing human infections and subdivided further into AI-IV and BI-IV, respectively [5, 6]. To date, only three studies have been conducted in Kenya mainly among residents of informal urban settings with results indicating that assemblage B and subassemblage BIV are the most common in both children and adults [7–9], but to our knowledge, no studies exist on molecular characterization of *G. duodenalis* genotypes in rural Kenya. In addition, studies have shown associations of assemblages A and B including their subassemblages with demographic, environmental, water potability, hygiene practices, contact with domestic animals, and clinical factors [6, 7, 10, 11]. Despite studies among children from informal settlements in Nairobi, Kenya, illustrating relationships of assemblage B and subassemblages BIII and IV with vomiting, abdominal pain, diarrhoea, and fever [7], no studies in rural settings demonstrate a possible link of *G. duodenalis* genotypes with potential sources of infection and intestinal manifestations. As such, this study set out to determine the assemblage and subassemblage of *G. duodenalis* and their association with sources, and gastrointestinal manifestations in a rural population from western Kenya.

## 2. Material and Methods

### 2.1. Study Area, Study Design, and Population

The study settings, design, patients, and sample collection are described in our previous publication [12]. Briefly, a total of 147 outpatients, age range of 3-73 years attending Busia County Referral Hospital, western Kenya, were enrolled in the study. Information on sociodemographic (gender, age, weight, education level, occupation, window and roof type, household size, history of eating outdoors, rural or periurban residence and travel history, contact with cattle, goat, sheep and poultry, dogs and/or cats, and clinical presentation with fever, headache, vomiting, bloating, stomach upset, abdominal pain, diarrhoea, and/or history of nitroimidazole use) were recorded using questionnaires. Stool specimens were collected from the patients and microscopically examined independently for *G. duodenalis* trophozoites and/or cysts by two experienced microscopists. *G. duodenalis*-positive stool samples were subjected to DNA extraction using QiAmp^®^ DNA stool mini kit (Qiagen, UK), according to the manufacturer's protocols with additional vortexing 5 minutes after mixing with glass beads before extraction [13]. The DNA-positive samples were genotyped independently for *G. duodenalis* assemblages and subassemblages by polymerase chain reaction-restriction fragment length polymorphism (RFLP) using primers at glutamate dehydrogenase (GDH) and triose-phosphate isomerase (TPI) gene loci as previously described [14].

### 2.2. Nested PCR-Amplification and RFLPs of Glutamate Dehydrogenase and Triose-Phosphate Isomerase Genes

DNA was extracted from faecal specimens as previously described [12] and used for the genotyping assays. Seminested PCR (nPCR) amplification at the *glutamate dehydrogenase* (GDH) gene locus HQ616623 exon IV generated an ∼432 bp fragment using primers: GDHeF: 5′-TCAACGTYAAYCGYGGYTTCCGT-3′ for primary reaction and GDHiR: 5′-GTTRTCCTTGCACATCTCC-3′ and GDHiF: 5′-CAGTACAACTCYGCTCTCGG-3′ for secondary amplification. The reaction was performed in 25 *μ*l volume as previously described [15]. The amplified products were digested using two restriction enzymes: *NIa IV* (New England Biolabs, USA) and *RSaI* (New England Biolabs, USA) for assemblage and subassemblage discrimination. Seminested PCR reaction was performed as previously described [16]. *Giardia triose-phosphate isomerase* (TPI) gene at HQ179643 loci on exon III was amplified using the primers AL3543 5′-AAATIATGCCTGCTCGTCG-3′ and AL3546: 5′-CAAACCTTITCCGCAAACC-3′ for the first PCR reaction into 605 bp template, followed by further amplification by the primers AL3544: 5′-CCCTTCATCGGIGGTAACTT-3′ and AL3545: 5′-GTGGCCACCACICCCGTGCC-3′ generating a 532 bp product. The amplicons were digested with 1.0 units of endonucleases *BbvI*, *RsaI*, and *MnlI* (New England Biolabs Inc., USA) for assemblage and subassemblage differentiation. The digested products were resolved by electrophoresis (subcell model 192 electrophoresis system, Bio-Rad, USA) at 100 volts for 45 minutes at room temperature in 2% agarose gels (Invitrogen, USA) stained with 0.5 mg/ml ethidium bromide. The resolved fragments were visualized under UV light using the gel documentation system (Uvitec, UK) and compared the band size against positive and negative internal controls (New England Biolabs, USA).

### 2.3. Data Analysis

Data was analyzed using the statistical package for social sciences (IBM^®^ SPSS Statistics for Windows, Version 25.0. Armonk, NY: IBM Corp., USA). The distribution and associations of *G. duodenalis* genotypes with sources and risk factors, including intestinal manifestation of the infection, were determined using Fisher's exact tests followed by binary logistic regression. All tests were two-tailed, and a value of *p* < 0.05 was used for statistical inferences. Only data with risk and clinical factors associated with or without assemblage or subassemblage in question are presented in Table 1.

### 2.4. Ethical Approval

This study was conducted in accordance with the tenets of human subjects' research as outlined in the Helsinki Declaration [17]. Ethical approval was obtained from the Masinde Muliro University of Science and Technology Ethical Review Committee (Protocol: MU/403012-V36). The study was approved by the National Commission for Science Technology and Innovation (NACOSTI), and permission to collect samples was obtained from the Busia County Referral Hospital Management. Written informed consent and/or assent was obtained from each adult and parent/guardian of each child, respectively, prior to enrollment into the study. All individuals who were stool-positive for giardiasis received metronidazole treatment as per the Kenya Ministry of Health (MoH) guidelines for the treatment of intestinal parasites [18].

## 3. Results

### 3.1. *Giardia duodenalis* Assemblages and Subassemblages

The distribution of *Giardia duodenalis* assemblages and subassemblages is shown in Table 2. Of the 147 stool specimens analyzed from patients comprising of children (≥3 < 18 years; 27.9%) and adults (≥18 ≤ 73 years; 61.3%), 59.9% tested positive for *G. duodenalis*, either trophozoite or cyst form under microscopic examination. The *G. duodenalis*-positive isolates underwent successful DNA extraction and genotyping by PCR-RFLPs at the GDH or TPI gene loci. Overall, most of the assemblages were B assemblages (63.6%) as well as at TPI (61.4%) and GDH (68.8%) genotyping. Assemblage rates were also similar (overall, 20.5%; TPI, 21.3%; and GDH, 23.4%). However, mixed assemblages of A/B were higher in TPI (17.3%) relative to GDH (7.8%) genotypes. In relation to the subassemblage rates, BIII was the most prevalent (overall, 30.7%) and was higher on GDH (42.2%) genotyping compared to TPI (24.0%) genotyping. Other subassemblages in the specimens were overall (AI, 5.7%; AII, 8.0%; AIII, 3.4%; and BIV, 17.0%), TPI (AI, 5.3%; AII, 8.0%; AIII, 4.0%; and BIV, 18.7%), and GDH (AI, 7.8%; AII, 10.9; AIII, 3.1%; and BIV, 21.9%). Consistent with overall higher rates of the BIII subassemblages AII/BIII (15.9%) and BIII/BIV (15.9%), at TPI, AII/BIII (17.3%), and BIII/BI-IV (18.7%), GDH, AII/BIII (7.8%), and BIII/BIV (4.7%) were the commonest mixed assemblages. Other mixed assemblages included AI/AII (1.1%) and AI/AIII (2.3%) at both loci, AI/AII (1.3%) and AI/AIII (2.7%) at TPI, and AI/AII (1.6%) and AI/AIII (0.0%) at GDH single genotyping.

### 3.2. Associations of *G. duodenalis* Assemblages with Risk and Clinical Factors

Assessment of the association of *G. duodenalis* assemblages with risk and clinical factors is summarized in Table 1. Young age (<18 years) individuals (42.9% vs. 9.4%; *p* = 0.026; OR, 0.396; 95% CI, 0.152-0.934; *p* = 0.029) were linked with higher odds of assemblage A infection. Similarly, a history of eating at outdoor (66.1% vs. 35.0%; *p* = 0.043; OR, 1.568; 95% CI, 1.914-6.634; *p* = 0.044) is associated with increased odds of assemblage B infection. As for gastrointestinal symptoms, stomach upset (61.1% vs. 41.4%; *p* = 0.030; OR, 1.158; 95% CI, 1.031-4.798; *p* = 0.026) is associated with increased odds of assemblage A. Also, individuals who presented with vomiting (51.8% vs. 28.1%; *p* = 0.023; OR, 2.680; 95% CI, 1.220-8.652; *p* = 0.025) and steatorrhea (14.3% vs. 3.1%; *p* = 0.028; OR, 3.022; 95% CI, 1.580-8.472; *p* = 0.030) were likely to be infected with assemblage B infections. Similarly, individuals with a history of previous giardiasis treatment (50.0% vs. 25.7%; *p* = 0.045; OR, 1.309; 95% CI, 1.093-7.011; *p* = 0.046) were likely to be infected with assemblage A infection. In relation to anthropozoonotic risk, contact with (55.6% vs. 25.7%; *p* = 0.047; OR, 1.334; 95% CI, 1.156-6.691; *p* = 0.046) and (37.5% vs. 19.6%; *p* = 0.034; OR, 1.795; 95% CI, 1.677-4.756; *p* = 0.036) was linked with higher odds of assemblages A and B infections, respectively. Likewise, contact with goats (16.1% vs. 9.4%; *p* = 0.029; OR, 1.538; 95% CI, 1.777-8.671; *p* = 0.030) and poultry in the homestead (55.4% vs. 46.9%; *p* = 0.042; OR, 1.778; 95% CI, 1.256-7.360; *p* = 0.045) was associated with increased odds of assemblage B infection.

Consistent with assemblages B, gastrointestinal symptoms, steatorrhea stool (66.7% vs. 6.8%; *p* = 0.037; OR, 1.510; 95% CI, 1.058-8.444; *p* = 0.042) was associated with higher odds of subassemblage BIV infection. Similarly, previous giardiasis treatment (42.8% vs. 28.4%; *p* = 0.036; OR, 1.319; 95% CI, 1.050-6.046; *p* = 0.038) was linked with higher odds of subassemblage AII infection. Anthropozoonotic analysis showed that contact with cattle (66.0% vs. 50.6%;*p* = 0.034; OR, 1.793; 95% CI, 1.069-9.145;*p* = 0.043) and (33.3% vs. 19.7%;*p* = 0.025; OR, 2.364; 95% CI, 1.726-7.696;*p* = 0.033) was linked with subassemblage AI and BIII infections, respectively. Contact with poultry keeping (370% vs. 24.6%; *p* = 0.031; OR, 1.528; 95% CI, 1.161-8.790; *p* = 0.032) is associated with higher odds of subassemblage BIII infection.

## 4. Discussion

Although *G. duodenalis* genotypes can cause varying degrees of disease severity and mortality, the sources including transmission dynamics, as well as the gastrointestinal manifestations, are poorly characterized in rural communities with a high burden of the disease. Therefore, the present study used a multilocus genotyping approach to determine the assemblages and subassemblages of *G. duodenalis* and their association with environmental, anthropozoonotic, and gastrointestinal features of the disease among residents of a rural population in western Kenya.

Using a multilocus genotyping strategy at the GDH and TPI genes showing higher proportions of assemblage B (63.6%), relative to the A (20.5%) and mixed A and B (15.9%) assemblages, suggests that the burden of giardiasis in this rural area of western Kenya largely results from *G. duodenalis* assemblage B infections. Besides, assemblage A and mixed A and B assemblage infections further complicate the epidemiology of giardiasis in this study population. These results are consistent with previous multigene genotyping at GDH, TPI, and beta-giardin (BG) loci of DNA extracted from stool samples from symptomatic and asymptomatic Kenyan and Ugandan children illustrating a predominance of assemblage B over the A and mixed A and B assemblages [5–7, 9, 19]. Our study identified five subassemblages AI, AII, AIII, BIII, and BIV with subassemblages AII, BIII, and BIV being the most prevalent. These results are, in part, consistent with studies in children from Mozambique at GDH, TPI, and BG genes and Tanzania at ssu rDNA and GDH multilocus genotyping that showed similar rates of AII, BIII, and BIV [6, 11]. However, rates of AI and AIII differed from the studies in Mozambique and Tanzania using different multilocus genes [6, 11], and those in Zambia based on single GDH genotyping [20]. The variation in the genotyping results among studies can be explained by the differences in population studied, methods, targeted gene loci for molecular characterization, and geographic distribution of *Giardia* genetic variants. Our studies and Mozambican results were conducted in patients who were microscopy positive or had occult giardiasis with RFLP multilocus genotyping, whereas the Ugandan and Zambian studies enrolled asymptomatic children with sequencing genotyping assays [5, 20, 21]. Altogether, these results demonstrate the high genetic diversity in *G. duodenalis* isolates from African patients that is possibly related to differences in transmission patterns.

The results of the present study showing that contact with domestic animals is associated with higher odds of presenting with assemblage A, B, subassemblages AI or BIII *G. duodenalis* infections suggest an anthropozoonotic source of infection. To the best of our knowledge, this is the first study to show a possible link of freely roaming domestic animals with *G. duodenalis* assemblage and subassemblage transmission in a rural area of western Kenya. This finding is similar to previous detection of assemblage A and B in *G. duodenalis* isolates from cattle in Egypt, Ethiopia, and Tanzania [11, 22, 23], as well as from goats, ducks, and chicken in Côte d'Ivoire, Nigeria, and Tanzania [11, 24, 25]. In addition, studies in Tanzania detected BIV subassemblage infection in goats and Zebu cattle [11], while the subassemblages BI and BIII were previously detected from indigenous goats in Nigeria [25]. Furthermore, previous studies diagnosed subassemblages AI and AII from cattle in Egypt and Ethiopia [22, 23]. Overall, domestic animals form an important link in the transmission of giardiasis possibly through environmental, drinking water, and foodstuff contamination with their faecal matter.

The association of age < 18 years with assemblage B suggests that a younger age is a risk factor for the *G. duodenalis* assemblage B infection. This assertion is similar to previous studies in Uganda and Ethiopia showing that children aged 25-36 months and 6-9 years, respectively, were more likely to have *G. duodenalis* infection [26, 27]. Furthermore, studies in Mozambique demonstrated that assemblage B of the parasite accounted for 90% of giardiasis infection in children under five years of age [6]. Similar results were also obtained in asymptomatic Nigerian children 5 to 17 years old showing that assemblage B was more frequent than assemblage A infections [28]. The reasons as to why children appear to be more vulnerable to assemblage B infection include the fact that they are more active and thus likely to interact with contaminated environment and associated risk factors. In addition, the predominance of assemblage B in relation to A and B in children may be related to the predominance of this genetic variant in the environment. Indeed, studies in Haiti illustrated that children ≤ 5 years frequently engaged in practices and behaviors that lead to increased risk of enteric illnesses. These included playing in public areas; touching latrines, open drainage canals, animals, and their faeces; touching and mouthing food and objects; eating soil; and drinking surface water [29]. In particular, given the anthropozoonotic nature of assemblage B, and the low hygienic knowledge, attitudes, and practices associated with close touching and playing with each other, animals, the environment, and water bodies appear to promote assemblage B giardiasis among children from this rural region.

Similarly, a history of eating from outdoor including food vendors was associated with higher odds of having assemblage B infection, suggesting increased exposure to contaminated food and water as well as infected food handler and poor hygiene practices [30]. This proposition is consistent with previous studies among Ghanaian pregnant women indicating that intestinal parasitic infections frequently occurred in those consuming cooked food from vendors [31]. In addition, previous studies illustrated that high rates of giardiasis were linked to outside eateries and unscreened food handlers in Cameroon and Nigeria [32, 33]. In particular, assemblage B infection was associated with eating from restaurants and food handlers in Iran [34]. The increase in the risk of assemblage B infection, in part, can be explained by previous studies among food handlers in Nairobi and Kisii municipalities showing that they are likely to transmit this protozoan parasite to consumers via contaminated food and water [35, 36]. Altogether, these studies illustrate that assemblage B giardiasis infection is largely an anthroponotic infection with transmission from infected food handlers and contaminated eateries.

The results showing an association of *G. duodenalis* assemblage A and B infection with gastrointestinal symptoms of stomach upset, vomiting, and steatorrhea suggest an acute and chronic infection, respectively. These findings mirror our previous studies indicating that giardiasis in this rural population presents with gastrointestinal manifestations [12]. Likewise, these results are also, in part, similar to previous studies undertaken among symptomatic children in Kenya and Egypt showing that assemblage A and B infections manifest with fever, flatulence, stomach upset, diarrhoea, vomiting, and abdominal pain [7, 37]. In addition, higher rates of assemblage A infections were reported in symptomatic children complaining of diarrhoea and stomach upset in upper Egypt and Malaysia [38, 39]. Interestingly, in the present study, assemblage B and subassemblage BIV were associated with steatorrhea suggesting a more severe or chronic infection resulting from these genotypes. This observation is consistent with previous studies in symptomatic cases in Rwanda illustrating that children with chronic giardiasis were likely to present with low body weight and steatorrhea [19]. Altogether, these results imply that *G. duodenalis* assemblage B is more pathogenic. This assertion is consistent with the previous clinicopathological animal model and paediatric studies from Brazil demonstrating higher assemblage B pathogenicity characterized by higher parasite shedding, widespread mucosae damage, decreased brush-border enzyme function, and permeation of inflammatory cells into the intestinal mucosa [40, 41].

Assemblage A and, in particular, subassemblage AII was predominant among individuals with a history of 5-nitroimidazole treatment, suggesting either treatment failure or suboptimal dose and/or drug resistance. This, in part, may be related to drug resistance, over-the-counter drugs, nonadherence to optimal dosages, and concurrent use of alcohol which is antagonistic to commonly available drugs such as metronidazole and thus impairing desirable treatment outcomes. The results presented here are also in concordance with findings of previous reports in children from Brazil showing recirculation of assemblage A infections after metronidazole treatment [42, 43]. Furthermore, *in vitro* and animal model studies showed low susceptibility to nitroimidazoles and furazolidone in assemblage A isolates [44], while higher rates of metronidazole resistance including multidrug resistance to nitroimidazoles were observed among assemblage A isolates from the Czech Republic [45].

### 4.1. Limitations of the Study

It is worthy to note that this study recruited both children (age ≥ 3 < 18 years) and adults referred to the clinical laboratory for stool analysis, as such the *G. duodenalis* genotypes were associated with gastrointestinal complaints and stool characteristics. Although sequencing techniques have been utilized in the molecular characterization of *G. duodenalis* genotypes [9, 28, 46, 47], the current study used the frequently applied multilocus genotyping strategy based on the constitutively expressed genetic markers GDH and TPI [48]. Despite the current study showing a link of *G. duodenalis* assemblages A and B with human contact with cattle, goats, and poultry, the rates of giardiasis in these animals including abattoirs and sewage treatment plants were not determined. Furthermore, information regarding prior fortnight nitroimidazole use was based on the recall response from the patients, but dosages including adherence and treatment schedule and concurrent alcohol use were not available to validate the effect of prior treatment on types and rates of *G. duodenalis* assemblages and subassemblages in the community.

## 5. Conclusion

This study provides insights into the *G. duodenalis* assemblages and subassemblages, and the related risk factors in rural western Kenya. In order to reduce giardiasis in this rural community, it is important to design integrated multifaceted interventions targeting decreasing environmental, anthroponotic, and anthropozoonotic contamination.

## Figures and Tables

**Table 1:** Associations of *G. duodenalis* assemblages and subassemblages with risk and clinical factors.

Characteristic	Giardia assemblage and subassemblages		Total cases	*p*	OR	95% CI	^∗^ *p*
	No, *n* (%)	Yes, *n* (%)					
*Assemblage*							
A, *n* = 18							
Stomach upset	29 (41.4)	11 (61.1)	40	**0.030**	1.158	1.031-4.798	**0.026**
Previous giardiasis treatment	18 (25.7)	9 (50.0)	27	**0.045**	1.309	1.093-7.011	**0.046**
Cattle	18 (25.7)	10 (55.6)	28	**0.044**	1.334	1.156-6.691	**0.045**
B, *n* = 56							
Age, <18 years	3 (9.4)	24 (42.9)	27	**0.026**	1.416	1.174-5.954	**0.029**
History of eating outdoor	11 (34.4)	37 (66.1)	48	**0.043**	1.568	1.914-6.634	**0.044**
Steatorrhea	1 (3.1)	8 (14.3)	9	**0.028**	3.022	1.580-8.472	**0.030**
Vomiting	9 (28.1)	29 (51.8)	38	**0.023**	2.680	1.220-8.652	**0.025**
Cattle	6 (18.8)	21 (37.5)	27	**0.034**	1.795	1.677-4.756	**0.036**
Goat	3 (9.4)	9 (16.1)	12	**0.029**	1.538	1.777-8.671	**0.030**
Poultry	15 (46.9)	31 (55.4)	46	**0.042**	1.778	1.256-7.360	**0.045**
*Subassemblage*							
AI, *n* = 5							
Cattle	42 (50.6)	3 (60.0)	45	**0.034**	1.793	1.069-9.145	**0.043**
AII, *n* = 7							
Previous giardiasis treatment	23 (28.4)	3 (42.8)	26	**0.036**	1.319	1.050-6.046	**0.038**
BIII, *n* = 27							
Cattle	12 (19.7)	9 (33.3)	21	**0.025**	2.364	1.726-7.696	**0.033**
Poultry	15 (24.6)	10 (37.0)	25	**0.031**	1.528	1.161-8.790	**0.032**
BIV, *n* = 15							
Steatorrhea	5 (6.8)	10 (66.7)	15	**0.037**	1.510	1.058-8.444	**0.042**

Results are number (*n*) and proportion (%) of patients. OR: odds ratio; 95% confidence interval: CI. The total assemblages and subassemblages from both genes (*glutamate dehydrogenase* and *triose-phosphate isomerase*) were used in determining the association. *p*: Fisher's exact tests; ^∗^*p*: logistic regression analysis. OR values > 1 indicate the odds of presence of positive association while values < 1 indicate the odds of absence of giardiasis (negative association). Significant *p* values are in bold.

**Table 2:** *G. duodenalis* assemblages and subassemblages.

	GDH, *n* (%)	TPI, *n* (%)	Total
Assemblages			
A	15 (23.4)	16 (21.3)	18 (20.5)
B	44 (68.8)	46 (61.4)	56 (63.6)
A/B	5 (7.8)	13 (17.3)	14 (15.9)
Subassemblages			
AI	5 (7.8)	4 (5.3)	5 (5.7)
AII	7 (10.9)	6 (8.0)	7 (8.0)
AIII	2 (3.1)	3 (4.0)	3 (3.4)
BIII	27 (42.2)	18 (24.0)	27 (30.7)
BIV	14 (21.9)	14 (18.7)	15 (17.0)
Mixed subassemblages			
AI/AII	1 (1.6)	1 (1.3)	1 (1.1)
AI/AIII	0 (0.0)	2 (2.7)	2 (2.3)
AII/BIII	5 (7.8)	13 (17.3)	14 (15.9)
BIII/BIV	3 (4.7)	14 (18.7)	14 (15.9)

Results shown are number (*n*) and proportion of genotypes. Only successful *glutamate dehydrogenase* (GDH, *n* = 64) and *triose-phosphate isomerase* (TPI, *n* = 75) genotyping were used in calculating frequencies for the assemblages and subassemblages.

## Data Availability

The data used to support the findings of the current study are available from the corresponding author upon request.
